# Tuberculosis in a Boar

**Published:** 1891-07

**Authors:** M. S. Mayo

**Affiliations:** Manhattan, Kansas


### TUBERCULAR ORCHITIS IN A BOAR.

M. S. MAYO, M. S., D. V. S.
MANHATTAN, KANSAS.

About the 15th of May, Dr. Orr left at my laboratory the testicle of a full-blooded Berkshire boar which had the appearance of being tuberculous, a microscopic examination confirming the diagnosis. Tuberculosis among swine is quite rare. Among 30,922 slaughtered at Augsburg, only three were affected.

